# Total knee arthroplasty in patients with Ranawat type-II valgus arthritic knee with a marked coronal femoral bowing deformity: comparison between computer-assisted surgery and intra-articular resection

**DOI:** 10.1186/s13018-016-0422-x

**Published:** 2016-08-03

**Authors:** Tsan-Wen Huang, Po-Yao Chuang, Chien-Yin Lee, Shih-Jie Lin, Kuo-Chin Huang, Shih-Hsun Shen, Yao-Hung Tsai, Mel S. Lee, Robert Wen-Wei Hsu

**Affiliations:** 1 Chang Gung Memorial Hospital, Chiayi, Taiwan; 2 Kaohsiung Chang Gung Memorial Hospital, Kaohsiung, Taiwan; 3 Chang Gung University, Taoyuan, Taiwan

**Keywords:** Bowing deformity of femur, Total knee arthroplasty, Genu valgus deformity, Computer-assisted surgery, Intra-articular bone resection

## Abstract

**Background:**

Proper limb and component alignments as well as soft tissue balance are vital for the longevity and optimal long-term outcomes of total knee arthroplasty (TKA). This procedure is technically demanding in patients with Ranawat type-II valgus arthritic knees with marked coronal femoral bowing. Computer-assisted surgery (CAS) and intra-articular bone resection with TKA are the treatments of choice for patients with ipsilateral extra-articular deformity. In theory, both CAS and intra-articular bone resection are beneficial in Ranawat type-II valgus arthritic knees with marked coronal femoral bowing deformity, but the literature on this topic is sparse. We compared the benefits of using these two techniques for TKA under this circumstance.

**Methods:**

Patients who had Ranawat type-II valgus arthritic knees and marked coronal femoral bowing deformity and had undergone TKA at our hospital between 2005 and 2013 were enrolled in this retrospective study. Patients treated with CAS were assigned to the CAS-TKA group; patients treated with intra-articular bone resection were assigned to the Bone-Resect-TKA group. Radiographic parameters and clinical outcomes (International Knee Society (IKS) scores and patellar scores) in both groups were compared.

**Results:**

Forty-seven patients (50 knees) met the inclusion criteria: 22 knees in the CAS-TKA group and 28 knees in the Bone-Resect-TKA group. Lateral retinaculum release was significantly (*P* = 0.008) higher in the Bone-Resect-TKA group. The joint-line was significantly properly restored in the CAS-TKA group (*P* = 0.011). The reconstructed mechanical axis was significantly (*P* = 0.012) closer to normal in the CAS-TKA group than in the Bone-Resect-TKA group. For component alignment, the femoral valgus and femoral flexion angles were significantly better in the CAS-TKA group (*P* = 0.002 and *P* = 0.006, respectively), but not the tibial valgus, tibial flexion, or patellar tilting angles. IKS scores and patellar scores were not significantly different between groups at a mean follow-up of 60.2 months.

**Conclusions:**

CAS-TKA was effective for obtaining proper alignment and joint-line restoration in patients with Ranawat type-II valgus arthritic knees and marked coronal femoral bowing deformity, but not for yielding better clinical outcomes. Additional large-scale prospective randomized cohort studies with long-term follow-ups are necessary to make evidence-based recommendations.

## Background

Accurately restoring the mechanical axis (MA) of the limb, aligning components, and properly balancing soft tissue are vital for the long-term success of total knee arthroplasty (TKA) [1]. In most TKAs, alignments can be restored and soft tissue balanced using appropriate bone cuts and soft tissue releases. However, TKA becomes technically more challenging when an arthritic knee is associated with intra-articular and extra-articular deformities [2, 3].

TKA on Ranawat type-II valgus arthritic knees is a challenge for orthopedic surgeons because of its associated intra- and extra-articular bony abnormalities [4, 5]. When using a conventional system to guide alignment, the distortion of the bony canal and variations in femoral anatomy are likely to decrease accuracy and cause improper limb and component alignments [4–7]. Balancing the soft tissue in Ranawat type-II valgus arthritic knees is another challenge and might contribute to complications such as peroneal nerve palsy and patellar instability [6, 7]. Many surgeons find it difficult to correct a valgus deformity using a conventional alignment guiding system without also using a constrained implant [4, 5].

**Fig. 1:**
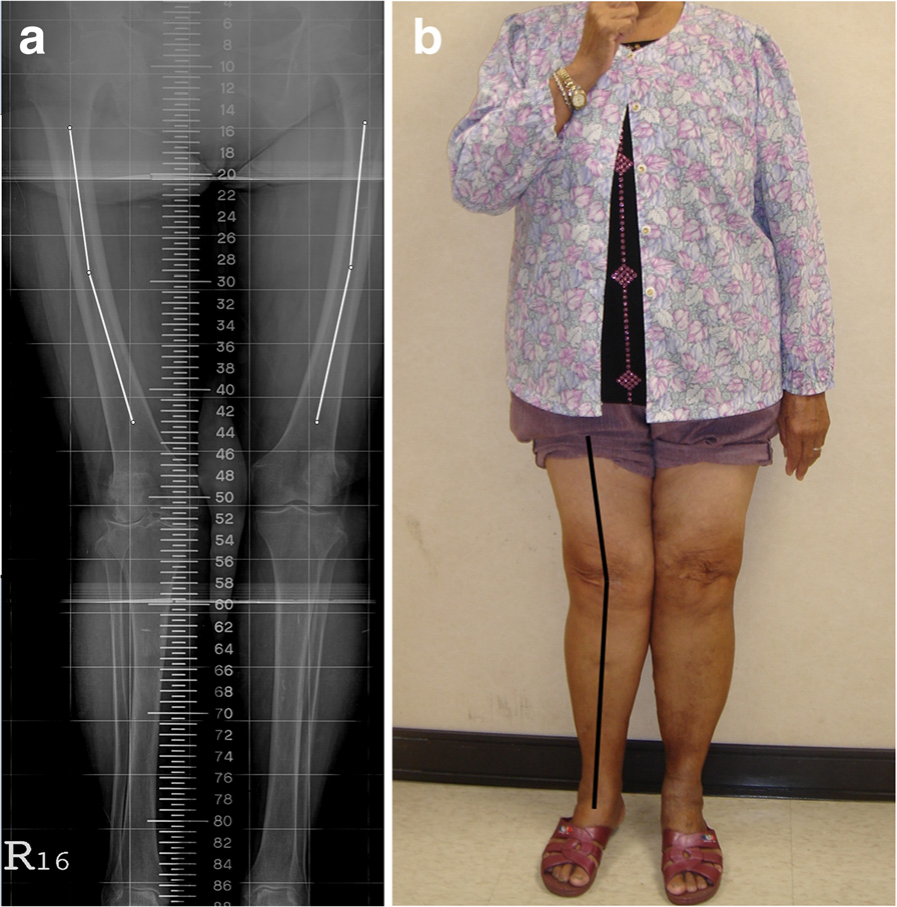
A 79-year-old woman with right-knee osteoarthritis and a genu valgus deformity of the right lower limb. **a** This preoperative full-length standing scanogram of the lower extremity shows a marked bilateral coronal femoral bowing deformity (measured using the method described in Mullaji et al. [21]). **b** Marked coronal femoral bowing deformities do not present clinically

Marked coronal femoral bowing deformity is easily missed. Evidence of this deformity cannot be seen in a short knee film, and it does not present clinically or intraoperatively (Fig. 1) [8, 9]. A rather high prevalence has been reported in China, India, Japan, Korea, Singapore, Taiwan, and Turkey [9–11]. The marked coronal bowing deformity alters the relationship between the MA and anatomical axis (AA) of the femur, thereby affecting the postoperative MA and the placement of the femoral component [12–14].

Ranawat type-II valgus arthritic knee with marked coronal femoral bowing is an unusual and complex deformity. Associated intra- and extra-articular deformities, as well as a difficult sequential soft tissue balance, might decrease the incidence of satisfactory outcomes in patients with TKA. In patients with arthritic knee and ipsilateral extra-articular deformity, TKA using intra-articular bone resection [15, 16] and computer-assisted surgery (CAS) [17, 18] are reported effective for increasing the accuracy and reproducibility of limb and component alignment with fewer outliers. Theoretically, both CAS and intra-articular bone resection are beneficial in Ranawat type-II valgus arthritic knees with marked coronal femoral bowing deformity, but the literature on this topic is sparse. Thus, we hypothesized that CAS-TKA would be more efficacious than intra-articular bone resection-TKA, and we investigated their effect on the radiographic and clinical outcomes of TKA.

## Methods

### Demographics

All patients who underwent TKA at Chang Gung Memorial Hospital, Chiayi since 2002 were routinely enrolled in our arthroplasty registry. The cost of CAS is not reimbursed by Taiwan’s National Health Insurance program, so after a complete explanation of the merits and disadvantages of both CAS-TKA and conventional techniques, the patients can choose the type of surgery they want. We prospectively collected clinical data: age, gender, diagnosis, length of hospital stay, surgical methods used, tourniquet time, total blood loss, complications associated with each surgical method, and preoperative and postoperative radiographic and clinical functional assessments.

Patient records in our arthroplasty database were manually reviewed to identify those patients with Ranawat type-II valgus arthritic knee and marked coronal femoral bowing who underwent a primary TKA. To minimize surgeon-related confounding factors, all of the TKAs we selected had been performed by the same surgeon (R.W.-W.H.), who has extensive experience using both conventional mechanical guides and computer-assisted navigation. To minimize implant-related confounding factors, the P.F.C.® Sigma® Knee System (DePuy Synthes, Warsaw, IN, USA) was used with all TKAs. Those patients with (a) a minimum follow-up of <24 months, (b) an extra-articular deformity of the femur or tibia because of previous trauma or surgery, and (c) incomplete medical records, radiographic analyses, or clinical functional assessments were excluded.

The patients were then divided into two subgroups: the CAS-TKA group (treated with CAS-TKA) and the Bone-Resect-TKA group (treated with intra-articular bone resection).

### Assessments

Preoperative and postoperative examinations of all patients included anteroposterior (AP) and lateral radiographs of the knee, a skyline view of the patellofemoral joint, and a full-length standing scanogram of the lower extremity [19]. These radiographs were used to measure the preoperative and postoperative MAs, valgus correction angles, and coronal femoral bowing angles. The magnitude of coronal femoral bowing was measured using the method previously described [8, 20]; femoral bowing greater than 5° was considered substantial [8, 20] (Fig. 1). The lateral patellar tilt and displacement were measured using the method described in Laurin et al. [21, 22]. The postoperative patellar tilting angle was measured using the method previously described [23]. The position of the prosthetic joint line was measured using the method described in Figgie et al. [24] on radiographs taken before and after surgery. An adequately restored joint line was defined as a joint line within 5 mm of the normal position [25, 26]. The component alignment angles—femoral valgus (FV) angle, tibial valgus (TV) angle, femoral flexion (FF) angle, and tibial flexion (TF) angle—were then measured [27]. The desired FV angle was based on the valgus correction angle of the distal femur, which was measured on the full-length standing scanogram of the lower extremity. The planned position was a TV angle of 90° in the coronal plane and a FF angle of 0° and TF angle of 87° in the sagittal plane. The goal was to reconstruct the MA and component alignments to within 3° of the proper position [28, 29]. The radiographs were evaluated by an independent assessor blinded to the patients’ demographic data. Intra-observer reliability, rated as “good” to “very good”, was assessed using the method described previously [30].

Clinical outcomes were assessed using International Knee Society (IKS) scores [31] and the patellar score [32], which were checked preoperatively and at the last follow-up. The active maximum range of motion (ROM) of the knee was measured with a goniometer. All clinical outcomes were collected and analyzed by two independent surgeons who were blinded to the surgical techniques used, the group to which each patient had been assigned, and the patients’ demographic data.

### Surgical technique

All TKAs were done using an anterior midline longitudinal skin incision and a medial parapatellar arthrotomy. After osteophytes had been removed from the femur, the soft tissue of the lateral compartment was released from the proximal tibia. In the Bone-Resect-TKA group, an intramedullary (IM) alignment guidance system was used for femoral preparation and an extramedullary (EM) guidance system was used for tibial preparation. The intra-articular bone resection technique previously described [15, 16] was used. After the distal femoral and tibial resection had been done, the knee was placed in full extension and a spacer block was used to determine the extension gap, the appropriate thickness of the tibial insert, and the coronal alignment of the lower limb, and to validate the soft tissue balance. The anatomical landmarks were precisely identified for femoral chamfer cuts, and a surgical transepicondylar line was drawn to judge femoral rotation. Whiteside’s line, the posterior condylar line, and the tibial cutting plane were supplemental in judging femoral rotation. After completing the osseous cuts of the femur, we placed the knee in 90° of flexion and a spacer block was used to evaluate the flexion gap. The rotation of the tibial component was adjusted, making it parallel to the axis between the medial-third of the tibial tuberosity and the center of the tibial plateau. The posterior cruciate ligament (PCL) was evaluated using the pull-out lift-off test previously described [33]. The PCL was then recessed as needed from its insertion site in the tibia. The soft tissue balance was assessed in a trial reduction and achieved by sequentially releasing the tight structures, as recommended [34]. The tourniquet was then deflated, and the hemostasis and patellar tracking were assessed using the no-thumb test.

In the CAS-TKA group, the prostheses were implanted with the assistance of a computer tomography (CT)-free navigation system (BrainLAB, Munich, Germany). After the knee joint had been exposed, an adequate amount of synovium was removed to render registration precise. All anatomic landmarks were identified and then sequentially registered in the navigation system. The implant size and orientation were identified by dragging the pointer along the bone surface to reconstruct the three-dimensional bone model. Then, femoral preparation followed by tibial preparation was completed under the navigation. The femoral component was referenced parallel to the anterior cortex of the distal femur and the transepicondylar line. After completing the osseous cuts of the femur and tibia, PCL tension was assessed using the method previously described [33]. The soft tissue balance was assessed in a trial reduction, and proper balance was achieved by sequentially releasing the tight structures using real-time and quantitative feedback from the navigation [34]. The tourniquet was then deflated, hemostasis done, and patellar tracking assessed.

All patients enrolled in this study were treated with the same protocol, which included the use of wound suction drains for 48 h and a continuous passive motion machine for passive ROM exercises four times daily (30 min per exercise session) from the day of surgery until the day of discharge from the hospital. Under the supervision of a physical therapist, the patients started active knee-motion exercises and began standing at bedside or walking with crutches or a walker twice daily for 30-min sessions. All patients used crutches or a walker with full weight-bearing for 6 weeks and a cane when needed thereafter.

### Statistical analysis

All data were collected and independently entered into a Microsoft Excel spreadsheet by two independent surgeons who were blinded to the surgical techniques used and the group to which each patient had been assigned. After the spreadsheets had been rechecked for missing and illogical data, the data were copied into SPSS 13.0 for Windows (SPSS Inc., Chicago, IL, USA) and analyzed. A *χ*^2^ test and Fisher’s exact test were used to compare the quality of implantation (measured against the ideal position) between the two groups with these parameters. Student’s *t* test was used to compare the variables of age, body height, body weight, body mass index, hospital stay, tourniquet time, blood loss, difference in perioperative hemoglobin levels, follow-up time, functional results, and radiographic parameters. All data were analyzed by an independent statistician who was blinded to the surgical outcomes. Significance was set at *p* < 0.05.

## Results

### Demographic data

Forty-seven patients (mean age 70 years; range 63 to 86 years) (50 knees 22 knees in the CAS-TKA group and 28 in the Bone-Resect-TKA group) were enrolled in this study. Degenerative osteoarthritis was diagnosed in all 50 knees. The mean body height was 156 cm (range 133 to 174 cm), mean body weight 67 kg (range 35 to 95 kg), and mean body mass index was 27.4 kg/m^2^ (range 19.8 to 38.1 kg/m^2^). The mean follow-up time was 60.2 months (range 24 to 97 months). There were no significant demographic differences between the two patient groups (Table 1).

**Table 1:** Demographic data of the patients

Parameters	CAS-TKA	Bone-Resect-TKA	*P* value
	*n* = 22	*n* = 28	
Age (years)	70 (63–86)	71 (64–85)	0.895
Body height (cm)	156 (147–172)	154 (133–174)	0.716
Body weight (kg)	66 (47–79)	67 (35–95)	0.504
Body mass index (kg/m^2^)	26.8 (21.8–31.1)	27.1 (19.8–38.1)	0.324
Hospital stay (days)	6.6 (5–10)	6.3 (5–10)	0.114
Follow-up time (months)	61.5 (24–82)	59.5 (26–97)	0.148

Data are mean (range). *P* for between-group comparisons was determined using *t* tests

*CAS-TKA* knees treated with computer-assisted surgery—total knee arthroplasty, *Bone-Resect-TKA* knees treated with intra-articular bone resection—total knee arthroplasty

Statistically significant (*P* < 0.05)

### Perioperative data

There was one significant perioperative difference between the two groups. To obtain adequate patellar tracking, 12 knees (42.9 %) in the Bone-Resect-TKA group, but only 2 knees (9.1 %) in the CAS-TKA group, required releasing the lateral retinaculum (*P* = 0.008). Total blood loss and perioperative hemoglobin levels were lower in the CAS-TKA group than those in the Bone-Resect-TKA group; however, the differences were not significant. There were no significant differences in the tourniquet time or the incidence of bone grafting (Table 2).

**Table 2:** Perioperative data

Parameters	CAS-TKA	Bone-Resect-TKA	*P* value
	*n* = 22	*n* = 28	
Perioperative data			
Total blood loss (ml)	601 (215–770)	762 (285–895)	0.071
Difference of perioperative hemoglobin level (g/dL)	1.1 (0.4–2.1)	1.8 (0.6–2.5)	0.112
Tourniquet time (minutes)	111 (68–117)	109 (72–129)	0.532
Lateral retinaculum for patellar tracking	2 (9.1 %)	12 (42.9 %)	0.008*
Bone grafting	10 (45.5 %)	12 (42.9 %)	0.540

Data are mean (range) or (%). *P* for between-group comparisons was determined using *χ* ^2^ tests for categorical variables and *t* tests for continuous variables

*CAS-TKA* knees treated with computer-assisted surgery—total knee arthroplasty, *Bone-Resect-TKA* knees treated with intra-articular bone resection—total knee arthroplasty

*Statistically significant (*p* < 0.05)

### Preoperative and postoperative lower limb alignment and component alignment

The preoperative MA between the two groups was not significantly different. The postoperative MA was significantly (*P* = 0.012) closer to normal in the CAS-TKA group than that in the Bone-Resect-TKA group. The postoperative MA was corrected to 180° (range 178 to 181°) in the former and 178° (range 176 to 184°) in the latter (Table 2). There was significantly (*P* = 0.011) less joint line elevation in the CAS-TKA group than that in the Bone-Resect-TKA group. For component alignment, the femoral valgus and femoral flexion angles were significantly (*P* = 0.002 and *P* = 0.006, respectively) better for the CAS-TKA group (Table 3). The percentage of procedures that had the ideal reconstructed MAs and component alignments was similar between the two groups (Table 4).

**Table 3:** Radiographic data

Parameters	CAS-TKA	Bone-Resect-TKA	*P* value
	*n* = 22	*n* = 28	
Radiographic data			
Preoperative MA (°)	193° (192°–198°)	194° (191°–196°)	0.954
Postoperative MA (°)	180° (178°–181°)	178° (176°–184°)	0.012*
Valgus correction angle of the distal femur (°)	10° (8°–12°)	10° (7°–12°)	0.739
Coronal femoral bowing angle (°)	8° (7°–13°)	9° (8°–12°)	0.714
Preoperative congruent angle (°)	12° (2°–38°)	12° (1°–39°)	0.887
Postoperative patellar tilting angle (°)	2° (1°–4°)	3° (2°–5°)	0.855
Joint line elevation (mm)	1 (−1–3)	3 (2–6)	0.011*
Component alignment			
Femoral valgus angle (°)	98° (97°–102°)	97° (95°–100°)	0.002*
Femoral flexion angle (°)	1° (0°–7°)	3° (0°–7°)	0.006*
Tibial valgus angle (°)	89° (88°–91°)	90° (89°–91°)	0.716
Tibial flexion angle (°)	88° (84°–90°)	87° (83°–91°)	0.643

Data are mean (range). *P* for between-group comparisons was determined using *t* tests

*CAS-TKA* knees treated with computer-assisted surgery—total knee arthroplasty, *Bone-Resect-TKA* knees treated with intra-articular bone resection—total knee arthroplasty, *MA* mechanical axis

*Statistically significant (*P* < 0.05)

**Table 4:** Comparison of percentage of postoperative lower limb alignment (within 3° deviation) and component alignment

Parameters	CAS-TKA	Bone-Resect-TKA	*P* value
	*n* = 22	*n* = 28	
Mechanical axis within 3° deviation			
	20 (90.9 %)	18 (64.3 %)	0.029*
Component positioning			
Femoral valgus angle	20 (90.9 %)	18 (64.3 %)	0.029*
Femoral flexion angle	19 (86.4 %)	17 (60.7 %)	0.044*
Tibial valgus angle	22 (100 %)	26 (92.9 %)	0.309
Tibial flexion angle	20 (90.9 %)	25 (89.3 %)	0.616

Data are n (%). *P* for between-group comparisons was determined using *χ* ^2^ tests

*CAS-TKA* knees treated with computer-assisted surgery—total knee arthroplasty, *Bone-Resect-TKA* knees treated with intra-articular bone resection—total knee arthroplasty

*Statistically significant (*P* < 0.05)

### Functional results

Clinically, the active ROM improved from 95° to 115° in the CAS-TKA group and from 99° to 115° in the Bone-Resect-TKA group. The mean patellar score improved postoperatively in both groups (from 16.3 to 26.9 in the CAS-TKA group and from 16.6 to 27.6 in the Bone-Resect-TKA group). On the IKS scoring system, the mean pain score improved from 16.3 to 46.7 in the CAS-TKA group and from 15.8 to 47.1 in the Bone-Resect-TKA group; the mean clinical knee score improved from 39.5 to 96.2 and from 39.1 to 97.6, respectively; and the mean functional knee score improved from 35.2 to 95.4 and from 34.3 to 96.6, respectively. Although there were marked improvements in all of the postoperative scores, there were no significant preoperative or postoperative differences between the two groups (Table 5).

**Table 5:** Preoperative and postoperative patellar, IKS, and ROM functional scores

Parameters	CAS-TKA	Bone-Resect-TKA	*P* value
	*n* = 22	*n* = 28	
Preoperative functional score			
Patellar score (points)	16.3 (10–24)	16.6 (10–24)	0.583
IKS pain score (points)	16.3 (10–20)	15.8 (10–20)	0.335
IKS clinical knee score (points)	39.5 (11–65)	39.1 (16–60)	0.714
IKS functional knee score (points)	35.2 (20–55)	34.3 (20–50)	0.504
Active range of motion (°)	95° (85°–120°)	99° (90°–120°)	0.960
Postoperative functional score			
Patellar score (points)	26.9 (20–30)	27.6 (21–30)	0.822
IKS pain score (points)	46.7 (40–50)	47.1 (40–50)	0.716
IKS clinical knee score (points)	96.2 (87–100)	97.6 (90–100)	0.887
IKS functional knee score (points)	95.4 (80–100)	96.6 (90–100)	0.668
Active range of motion (°)	115° (100°–25°)	115° (105°–125°)	0.541

Data are mean (range). *P* for between-group comparisons was determined using *t* tests

*CAS-TKA* knees treated with computer-assisted surgery—total knee arthroplasty, *Bone-Resect-TKA* knees treated with intra-articular bone resection—total knee arthroplasty, *IKS Score* International Knee Society Score

Statistically significant (*P* < 0.05)

### Complications

There were no complications in any of the 50 knees. Specifically, no patient required advancement of the medial collateral ligament (MCL) or was converted to a posterior-stabilized prosthesis or constrained components because of an excessively bony resection or inadequate soft tissue release during the operation. There were no cases of wound infection, peroneal nerve neurapraxia, pulmonary emboli, or deep vein thrombosis. No periprosthetic fractures, joint instability, or patellar tracking problems were encountered. No patients showed loosening or osteolysis on radiography at the time of the last follow-up, and no patients underwent revision surgery for any reason.

## Discussion

The most important finding of this study was that CAS was more efficacious than intra-articular resection for facilitating a properly reconstructed MA, femoral component placement, and restoration of the joint-line when performing TKA on a patient with a Ranawat type-II valgus arthritic knee with a marked coronal femoral bowing deformity. However, CAS did not yield a better clinical outcome at a mean follow-up of 60.2 months.

The long-term outcome of TKA depends on good component positioning and a reconstructed MA that is within 3° of neutral in the coronal plane [1–3]. However, TKA on either Ranawat type-II valgus arthritic knees or on knees with marked coronal femoral bowing deformities is technically demanding because associated intra-articular bony abnormalities (e.g., distal femoral hypoplasia, posterior femoral condylar erosion, and patellar maltracking) and extra-articular bony abnormalities (e.g., increased femoral neck-shaft angle and metaphyseal remodeling of both the femur and the tibia) might render the use of conventional guidance systems inadvisable [6, 7]. However, from an anatomical perspective, the angular relationship between the MA and AA of the femur is influenced by a marked bowing deformity in the coronal plane [8–11]. This anatomic feature is easily overlooked because it is neither clinically apparent nor evident on short-film radiographs of the knee [19] (Fig. 1).

When using conventional TKA, the choice of cutting block used for a distal femoral bone cut perpendicular to the MA of the femur depends upon the valgus correction angle of the distal femur. For valgus arthritic knees, Ranawat et al. [5] suggested using a 3° valgus distal femoral cutting block rather than the usual 5° to 7° cutting block. However, we found that the femoral bowing in patients with Ranawat type-II valgus arthritic knees with marked coronal femoral bowing deformities resulted in a more variable valgus correction angle of the distal femur (range 7° to 12°). Routine use of a 3° valgus distal femoral cut might not be advisable. Any femoral cutting system must incorporate appropriate preoperative planning to adjust the cuts to accommodate the deformity. However, the majority of currently available femoral jigs do not provide a broad enough choice of valgus cut angles to realize an ideal reconstructed MA in patients with such deformities [12–14].

**Fig. 2:**
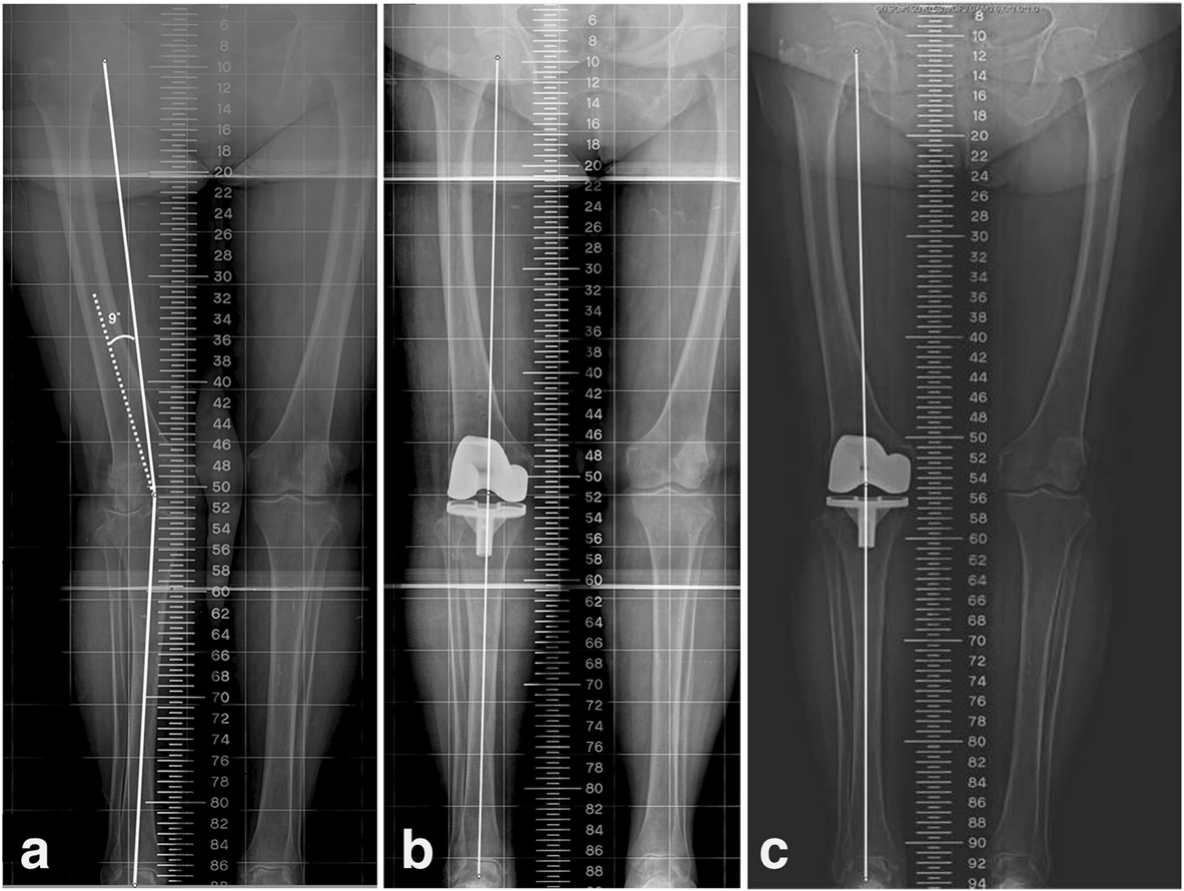
Representative results with one patient who had undergone CAS-TKA for Ranawat type-II valgus arthritic knees and marked coronal femoral bowing. **a** This preoperative full-length standing scanogram of the lower extremity shows Ranawat type-II valgus arthritic knees with a mechanical anatomical axis of 193 and a 9° femoral valgus resection angle. **b** A postoperative radiograph showing complete restoration of limb alignment after undergoing CAS-TKA. **c** Eight years later, the limb remains in excellent alignment

Staged and simultaneous corrective osteotomy and TKA have been advocated to achieve proper limb alignment and better ligament balancing when a patient has an arthritic knee with an extra-articular deformity [35–37]. However, this technique might be associated with substantial complications, including delayed union or nonunion at the osteotomy site, failure of internal fixation, infection of the osteotomy site, and arthrofibrosis [15]. Hadjicostas et al. [38] studied 15 patients with valgus deformities of 17° to 27° and reported excellent mid-term results using CAS to guide a simultaneous osteotomy of the lateral femoral condyle before TKA. The deformities in their patients were much larger in the coronal plane than were the deformities in our series (range 11° to 18°) and were, therefore, difficult to correct with intra-articular bone resection or CAS. In addition, if a deformity in the supracondylar area is ≥20°, soft tissue balancing will be difficult after joint line resection. Therefore, staged or simultaneous corrective osteotomy and TKA should be considered in patients with a large deformity [15]. In this study, we used a previously described intra-articular bone resection method [15, 16]. Use of an IM guidance system with a modified starting hole in the knee has been reported to be effective for arthritis of the knee with ipsilateral extra-articular deformity [15]. However, this method also increases the risk of improper postoperative MA and femoral component malalignment. The most likely reason is that the incomplete insertion of IM rods may result in a subsequent erroneous distal femur resection [3]. On the other hand, with CAS-TKA, the surgeon can focus on the centers of the hip, knee, and ankle joints and ignore any extra-articular deformities of the femur and tibia, which allows for cuts that can provide the desired reconstructed alignment of the limb and its components [9, 13, 14] (Fig. 2).

Malrotation of the femoral component affects patellofemoral tracking and might increase contact pressure, which leads to accelerated and excessive wear on the patellar button [39]. In Ranawat type-II valgus arthritic knees, the lateral femoral condyle deficiencies often render the posterior condylar axis inaccurate as a reference for determining femoral component rotation. A fixed 3° external rotation might not be suitable under this circumstance [4–7]. In the current study, we used the transepicondylar axis as a reference to judge femoral rotation. Whiteside’s line, the posterior condylar line, and the tibial cutting plane were supplemental in judging femoral rotation. However, significantly more knees in the Bone-Resect-TKA group (12 = 42.9 %) than in the CAS-TKA group (2 = 9.1 %) required release of the lateral retinaculum to obtain adequate patellar tracking. When using CAS-TKA, the surgeon can judge the accuracy of the cutting jig in the axial plane using real-time and quantitative feedback from the navigation. A mistake in visual judgment of a chamfer block in the axial plane might be the confounding factor that results in inadequate femoral rotation alignment in conventional TKA.

Balancing the soft tissue in Ranawat type-II valgus arthritic knees is another challenge [4, 5]. A precise osseous cutting technique can prevent a large extension gap and lax MCL. However, Ranawat type-II valgus arthritic knees have more medial soft tissue stretching and lateral soft tissue contraction, which might contribute to inappropriate soft tissue management. Over-releasing of soft tissue might result in component malalignment and an unplanned conversion to a constrained prosthesis [3–7]. Thick polyethylene tibial inserts have been used to compensate for excessive laxity, but this risks elevating the joint line and increasing the TKA failure rate [40]. Joint line elevation secondary to placing a thick polyethylene spacer increases the risk of peroneal nerve neuropraxia, and patellofemoral contact forces might contribute to postoperative complications such as pain, polyethylene wear, and inferior clinical results [41]. Limiting joint line elevation to <5 mm in primary TKAs is considered satisfactory because it does not affect clinical outcomes [26]. To overcome these difficulties, many soft tissue handling procedures have been used with consistent and satisfactory results [6]. However, the ideal soft tissue balance still largely depends upon the surgeon’s experience. Lack of a quantitative and objective monitoring system might reduce the incidence of satisfactory TKA outcomes in the hands of less experienced surgeons. With the real-time and quantitative feedback of a navigation system, surgeons can provide a proper soft tissue balance and prevent joint-line elevation. Ensini et al. [42] and Chou et al. [43] reported that the joint line is well restored and the risk of overstuffing is limited with CAS-TKA. We too found less joint line elevation with CAS-TKA than with conventional TKA.

This study has some limitations. First, it has all the inherent limitations and biases of a retrospective study. However, all patients were treated by the same experienced surgeon using the same protocol, which decreases the effects of some confounding factors. Second, this was a radiographic and short-term clinical follow-up study. Although the CAS-TKA group had higher percentages of ideal postoperative MAs and component alignment, CAS-TKA did not yield better clinical results than did Bone-Resect-TKA at a mean follow-up of 60.2 months. One of the reasons for not finding significant differences in clinical outcomes is the duration of follow-up. If the minimum follow-up period is more than 10 years, differences in failure rates between well-aligned and malaligned TKAs might become apparent. Additional long-term studies on CAS-TKA are needed to determine whether radiographic benefits result in better long-term clinical outcomes. Third, there were only 50 knees in this study, which reflects the relative rarity of Ranawat type-II valgus arthritic knees with marked coronal femoral bowing deformities in patients undergoing TKA. One study [44], using the IKS scoring system to calculate a sample size with a power of 80 % and a significance of 0.05 to detect a difference of 5 points in the IKS score (estimated SD of 8), found that 33 knees were required per group. With only 22 cases of CAS-TKA, our study was too underpowered to show significant differences with Bone-Resect-TKA. Although it is always better to have more patients in a prospective study to clarify the effect of CAS-TKA on proper alignment and clinical outcomes, it would be difficult to do a prospective randomized large-scale cohort study based on this rare deformity. Finally, the current study did not include CT-based navigation, which might have an accuracy advantage in rotational alignment because it allows accurate preoperative planning on patient-specific three-dimensional bone models. However, CT-based navigation generates more radiation and requires additional time for preoperative planning, and the scanning increases costs. After the anatomic landmarks have been adequately identified, image-free navigation might be sufficient for proper component alignment.

## Conclusions

Ranawat type-II valgus arthritic knee with marked coronal femoral bowing reduces the accuracy of restoring postoperative MA, femoral component alignment, and the joint-line elevation level when intra-articular resection is used. Instead of the traditional fixed valgus correction angle of the distal femur in Asian patients, the angle first needs to be determined and then adjusted using a full-length standing scanogram of the lower extremity. Modifying the femoral jig to provide a wider choice of valgus cut angles or using a staged or simultaneous femoral osteotomy combined with TKA are all viable options. Our data suggest that CAS-TKA can be an effective alternative for restoring the joint line, properly aligning the limb, and accurately positioning the components. However, we found no significant difference in clinical outcomes between the CAS-TKA and Bone-Resect-TKA groups. Studies with long-term follow-ups are needed to determine whether the improvement in radiographic results actually translates to better clinical outcomes.

## Abbreviations

AA, anatomical axis; CAS-TKA, computer-assisted surgery total knee arthroplasty; EM, extramedullary; FF, femoral flexion angle; FV, femoral valgus angle; IKS, International Knee Society; IM, intramedullary; MA, mechanical axis; ROM, range of motion; TV, tibial valgus angle; TF, tibial flexion angle; TKA, total knee arthroplasty
